# Time to consider doxycycline in the standard treatment of lymphatic filariasis? Emerging evidence on use of doxycycline as an adjunct to hygiene protocols

**DOI:** 10.1186/s41182-025-00726-4

**Published:** 2025-03-24

**Authors:** Mian Zahid Jan Kakakhel, Shree Rath, Maheen Sheraz, Diya Rathi, Raheel Ahmed

**Affiliations:** 1 Rehman Medical College, Peshawar, Khyber Pakhtunkhwa Pakistan; 2 All India Institute of Medical Sciences, Bhubaneswar, India; 3 Continental Medical College, Lahore, Pakistan; 4 Dow University of Health Sciences, Karachi, Pakistan; 5 National Heart and Lung Institute, Imperial College London, London, UK

**Keywords:** Doxycycline, Lymphatic filariasis, Hygiene protocols

## Abstract

With lymphatic filariasis being a rampant condition in tropical countries, multiple treatment modalities are being explored for their efficacy and practicability of use in low resource settings. Doxycycline, a commonly available drug across the world, has been proposed to improve patient status in those suffering with lymphatic filariasis. In conjunct with hygiene protocols, emerging trials have highlighted the success of doxycycline as an add-on drug. In this letter, we highlight the major findings in recent trials, and the scope of integrating doxycycline into national elimination strategies against filariasis.

Dear Editor,

Lymphatic filariasis (LF), commonly known as elephantiasis, is a chronic parasitic infection. Mosquitos are the main carriers for this parasitic infection, which predominantly affects humans. Prolonged infection can result in testicular tumors, hydrocele, and limb edema. As larvae mature into adult worms within the bloodstream, the body becomes more susceptible to recurrent infections due to lymphatic obstruction and impaired drainage [1]. In the late twentieth century, approximately 20–40 million individuals worldwide were affected by LF, with an additional 100 million infected but clinically asymptomatic, and up to one billion at risk of infection. The Global Programme to Eliminate LF aimed to eradicate the disease by 2020 [1, 2]. An update from this initiative in 2010 stated that it was halfway to eradicating filariasis, and by 2015, over 820 million individuals had received 6.2 billion treatments. Currently, 51.4 million people are expected to be affected by LF worldwide, with 40 million reporting LF-related morbidities [3].

Since there is no established drug treatment for LF, management is the primary approach to minimize or slow the progression of swelling and prevent related infections [4]. Hygiene-based care of LF mainly focuses on cleaning the affected limbs with suitable topical antibiotics and antifungals, which has been shown to effectively delay the progression of the disease. Antibiotics such as doxycycline can be used as an adjunct to hygiene protocols in managing LF [3]. According to a randomized controlled trial (RCT) conducted with 356 participants in Ghana found that, while the main approach to managing LF was preventive chemotherapy, combining doxycycline with strict hygiene protocol could successfully prevent acute attacks [5]. Another RCT conducted in Ghana revealed that patients treated with doxycycline experienced a significant reduction in lymphedema severity after 12–24 months, with 43.9% showing major improvement [6]. Similarly, a RCT conducted in Thailand, where a single dose of doxycycline was given, reported a significant reduction in the severity of adverse reactions compared to the placebo [7]. Furthermore, a double blind, randomized placebo-controlled trial in Tanzania reported that after the first 6 months of the treatment, the doxycycline group experienced significantly fewer acute adenolymphangitis attacks—which is a chronically disabling part of LF—compared to the placebo group. Hence, doxycycline plays a key role in halting the progression of LF [3].

One of the main benefits of using doxycycline is its widely available and cost-effective property, making it a viable choice for widespread use in endemic areas. Due to its high safety profile and mild side effects, especially in non-pregnant patients, doxycycline can be used more frequently. Moreover, there is no appreciable risk of resistance emerging, making doxycycline a dependable and long-lasting choice in the continuous battle against the illness [8].

There is a growing need to integrate doxycycline therapy into the management of LF, alongside broader public health efforts to improve hygiene practices and reduce disease transmission. When combined with hygiene interventions, such as proper care of affected limbs to prevent infections, doxycycline can help maintain cleaner conditions, reducing the risk of secondary infections, and improving patient outcomes. With mass drug administration (MDA) campaigns and public education on hygiene, doxycycline therapy offers a comprehensive approach to managing LF, not only mitigating its symptoms but also playing a pivotal role in the broader goal of disease elimination and long-term control.

**How would they implement the use of doxycycline during the MDA program? How often would they give it to the patient? if they would stratify the patients according to the severity of the disease?**

The MDA program consists of ivermectin or ivermectin plus albendazole, which targets adult worms directly and reduces microfilariae levels. However, doxycycline targets Wolbachia bacteria, the obligatory endosymbiotic bacteria on which filarial worms rely for their survival and reproduction within the human host. This makes doxycycline a more effective adjunct to other drugs [9]. Furthermore, the recommended dose of doxycycline is 200 mg/day for 6 weeks every other year, or even annually. It is primarily used for individual with LF at stages 1–3 [6, 9].

## Data Availability

All data is freely available online.
